# A winged-helix DNA-binding protein is essential for self-fertility during sexual development of the homothallic fungus *Fusarium graminearum*

**DOI:** 10.1128/msphere.00511-24

**Published:** 2024-08-27

**Authors:** Jiyeun Park, Hosung Jeon, Aram Hwangbo, Kyunghun Min, Jaeho Ko, Jung-Eun Kim, Sieun Kim, Ji Young Shin, Yong-Hwan Lee, Yin-Won Lee, Hokyoung Son

**Affiliations:** 1 Institute for Plant Sciences, University of Cologne, Cologne, Germany; 2 Department of Agricultural Biotechnology, Seoul National University, Seoul, South Korea; 3 Department of Plant Science, Gangneung-Wonju National University, Gangneung, South Korea; 4 Research Institute of Climate Change and Agriculture, National Institute of Horticultural and Herbal Science, Jeju, South Korea; 5 Horticultural and Herbal Crop Environment Division, National Institute of Horticultural and Herbal Science, Wanju, South Korea; 6 Honam National Institute of Biological Resources, Mokpo, South Korea; 7 Research Institute of Agriculture and Life Sciences, Seoul National University, Seoul, South Korea; 8 Interdisciplinary Programs in Agricultural Genomics, Seoul National University, Seoul, South Korea; 9 Center for Plant Microbiome Research, Seoul National University, Seoul, South Korea; 10 Plant Immunity Research Center, Seoul National University, Seoul, South Korea; 11 Plant Genomics and Breeding Institute, Seoul National University, Seoul, South Korea; University of Georgia, Athens, Georgia, USA

**Keywords:** *Fusarium graminearum*, sexual reproduction, winged-helix DNA-binding protein

## Abstract

Sexual reproduction is crucial for increasing the genetic diversity of populations and providing overwintering structures, such as perithecia and associated tissue, in the destructive plant pathogenic fungus *Fusarium graminearum*. While mating-type genes serve as master regulators in fungal sexual reproduction, the molecular mechanisms underlying this process remain elusive. Winged-helix DNA-binding proteins are key regulators of embryogenesis and cell differentiation in higher eukaryotes. These proteins are implicated in the morphogenesis and development of several fungal species. However, their involvement in sexual reproduction remains largely unexplored in *F. graminearum*. Here, we investigated the function of winged-helix DNA-binding proteins in vegetative growth, conidiation, and sexual reproduction, with a specific focus on the *FgWING27*, which is highly conserved among *Fusarium* species. Deletion of *FgWING27* resulted in an abnormal pattern characterized by a gradual increase in the expression of mating-type genes during sexual development, indicating its crucial role in the stage-specific genetic regulation of *MAT* genes in the late stages of sexual development. Furthermore, using chromatin immunoprecipitation followed by sequencing analysis, we identified *Fg17056* as a downstream gene of Fgwing27, which is essential for sexual reproduction. These findings underscore the significance of winged-helix DNA-binding proteins in fungal development and reproduction in *F. graminearum*, and highlight the pivotal role of Fgwing27 as a core genetic factor in the intricate genetic regulatory network governing sexual reproduction.

**IMPORTANCE:**

*Fusarium graminearum* is a devastating plant pathogenic fungus causing significant economic losses due to reduced crop yields. In Fusarium Head Blight epidemics, spores produced through sexual and asexual reproduction serve as inoculum, making it essential to understand the fungal reproduction process. Here, we focus on winged-helix DNA-binding proteins, which have been reported to play crucial roles in cell cycle regulation and differentiation, and address their requirement in the sexual reproduction of *F. graminearum*. Furthermore, we identified a highly conserved protein in *Fusarium* as a key factor in self-fertility, along with the discovery of its direct downstream genes. This provides crucial information for constructing the complex genetic regulatory network of sexual reproduction and significantly contribute to further research on sexual reproduction in *Fusarium* species.

## INTRODUCTION

*Fusarium graminearum* is a destructive plant pathogenic fungus that causes Fusarium head blight (FHB) on various cereal crops, such as wheat, barley, and maize (1). Infection with this fungus results in significant yield losses and grain quality deterioration (2). Additionally, grain contamination by mycotoxins poses a risk to humans and animals (3, 4). FHB persists over time, with consistent reports of damage worldwide. FHB epidemics are initiated through rain-splash or aerial dispersal of spores, including conidia and ascospores (5, 6). These spores, produced during asexual and sexual reproduction respectively, are integral to the life cycle of *F. graminearum* (7). Ascospores, originating from the overwintering structure “perithecia,” serve as primary sources of inoculum. Ascosporogenesis involves successive divisions, including meiosis and post-meiotic mitosis, which contribute to genetic diversity (8, 9). Therefore, understanding the molecular intricacies of sexual reproduction is crucial for achieving effective FHB control.

In filamentous ascomycetes, sexual developmental processes proceed through the development of ascogonia, peridium formation, and subsequent internal maturation. These mating processes are tightly regulated by two mating-type (*MAT*) loci: *MAT1-1* and *MAT1-2* (9, 10). *MAT1-1* comprises *MAT1-1-1*, *MAT1-1-2*, and *MAT1-1-3*, while *MAT1-2* includes *MAT1-2-1* and *MAT1-2-3* (11). Functional studies on these genes have identified Mat1-1-1 and Mat1-2-1 as master regulators that control downstream genes required for sexual reproduction (12). In the homothallic fungus *F. graminearum*, both loci reside within a single nucleus, and transcriptomic analyses have implicated their roles in regulating genes associated with cell wall organization, fertilization, and development (13). However, the specific genetic factors required for sexual reproduction remain elusive, underscoring the importance of elucidating stage-specific gene regulation during sexual development.

Winged-helix DNA-binding proteins are found in both eukaryotes and prokaryotes, and play diverse roles in cellular processes (14). The winged-helix domain comprises two large loops (known as wings), three α-helices, and three β-sheets (15). Protein-DNA binding typically involves the recognition of specific DNA sites in the major groove by the α-helix, stabilized by the wings (16). Numerous proteins containing winged-helix DNA-binding domains regulate cell differentiation, development, and the cell cycle (17). For example, forkhead transcription factors, which possess a winged-helix DNA-binding motif, regulate mitotic genes (18, 19). Additionally, the Regulatory Factor X (RFX) family, a subclass of winged-helix DNA-binding proteins, functions in morphogenesis, nuclear division, and the regulation of genes related to ciliogenesis in eukaryotes (20, 21). However, the function of winged-helix DNA-binding proteins in fungal development remains incompletely explored in *F. graminearum*.

Our previous research identified 28 putative winged-helix DNA-binding proteins in *F. graminearum*, of which 10 were found to be involved in sexual reproduction through phenotyping (22). In this study, we investigate the necessity of these 10 winged-helix DNA-binding proteins in various processes associated with sexual reproduction. Based on their expression patterns during the sexual reproduction process, we propose a stage-specific regulatory mechanism for the expression of mating-type genes. This study enhances our understanding of the role of winged-helix DNA-binding proteins in sexual reproduction and contributes to elucidating the regulatory network governing sexual reproduction in *F. graminearum*.

## RESULTS

### Conservation of winged-helix DNA-binding proteins related to sexual reproduction in fungal species

**Fig 1:**
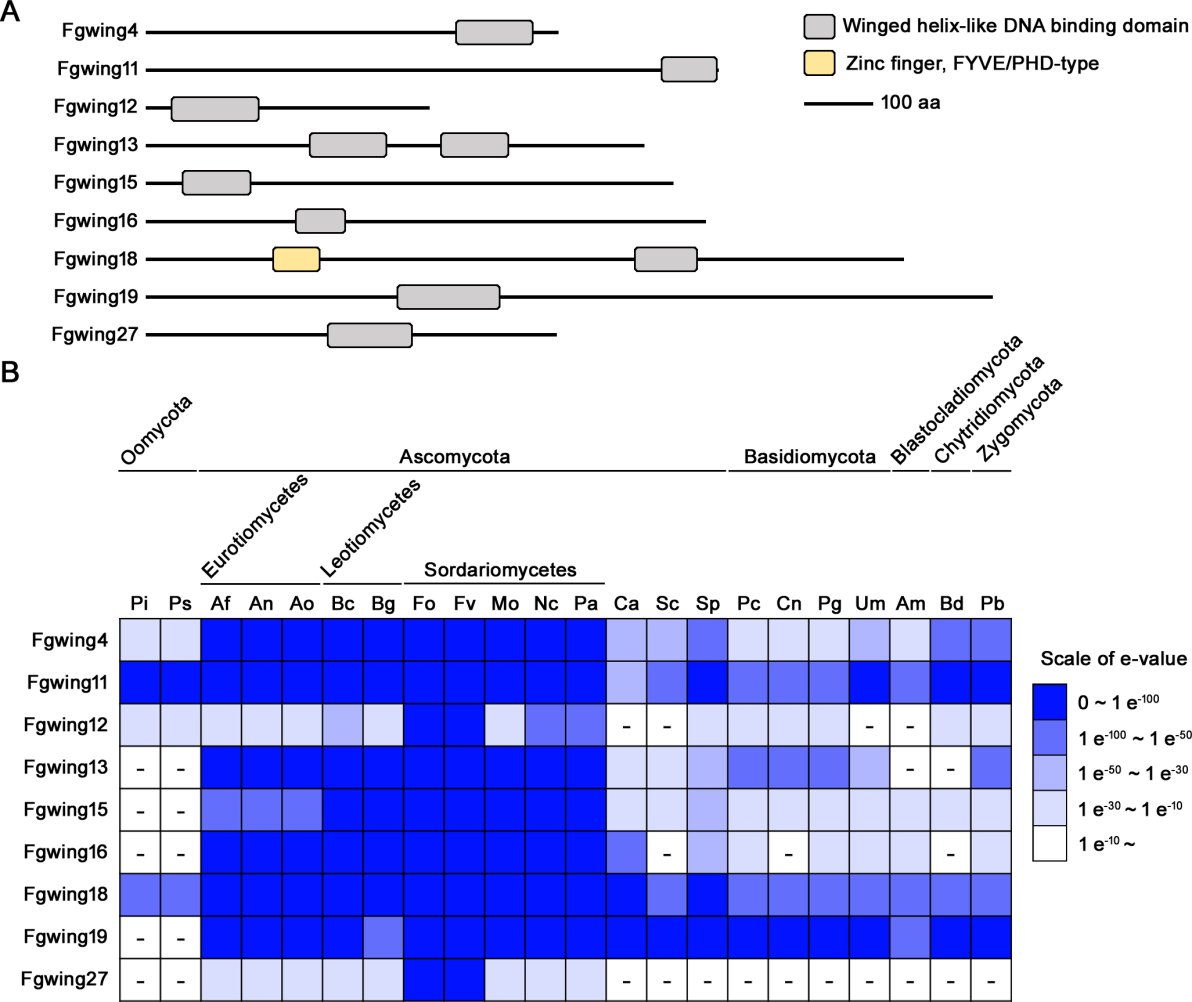
Presentation of the domain structure and conservation analysis of nine winged-helix DNA-binding proteins in *F. graminearum* (**A**) Protein domain architectures of putative sexual reproduction-related winged-helix DNA-binding proteins in *F. graminearum* (**B**) Conservation analysis of nine winged-helix DNA-binding proteins among 22 different species using the Comparative Fungal Genomics Platform (CFGP, http://cfgp.riceblast.snu.ac.kr).

We retrieved the protein sequences of the 10 putative winged-helix DNA-binding proteins and performed domain analysis using the InterPro database. This analysis revealed that nine of these proteins contain a winged-helix-like DNA binding domain (IPR036388): Fgwing4, Fgwing11, Fgwing12, Fgwing13, Fgwing15, Fgwing16, Fgwing18, Fgwing19, and Fgwing27 (Table S1). Fgwing13 harbors two winged-helix domains, whereas Fgwing18 possesses another putative DNA binding domain, the FYVE/PHD-type zinc finger (IPR011011) (Fig. 1A). We analyzed the conservation of these proteins across fungal species and related eukaryotic kingdoms using BLASTMatrix analysis. Most winged-helix DNA-binding proteins associated with sexual reproduction are well conserved within the subphylum Pezizomycotina, which includes the classes Eurotiomycetes, Leotiomycetes, and Sordariomycetes (Fig. 1B). Fgwing12 is also conserved across fungi, showing particularly high sequence similarity to *Fusarium* species. Interestingly, homologs of Fgwing27 are only detected in the subphylum Pezizomycotina and exhibit low similarity except in *Fusarium* species, suggesting that Fgwing27 may be particularly relevant to *Fusarium*-specific functions.

### Eight winged-helix DNA-binding proteins are responsible for sexual reproduction in *F. graminearum*

**Fig 2:**
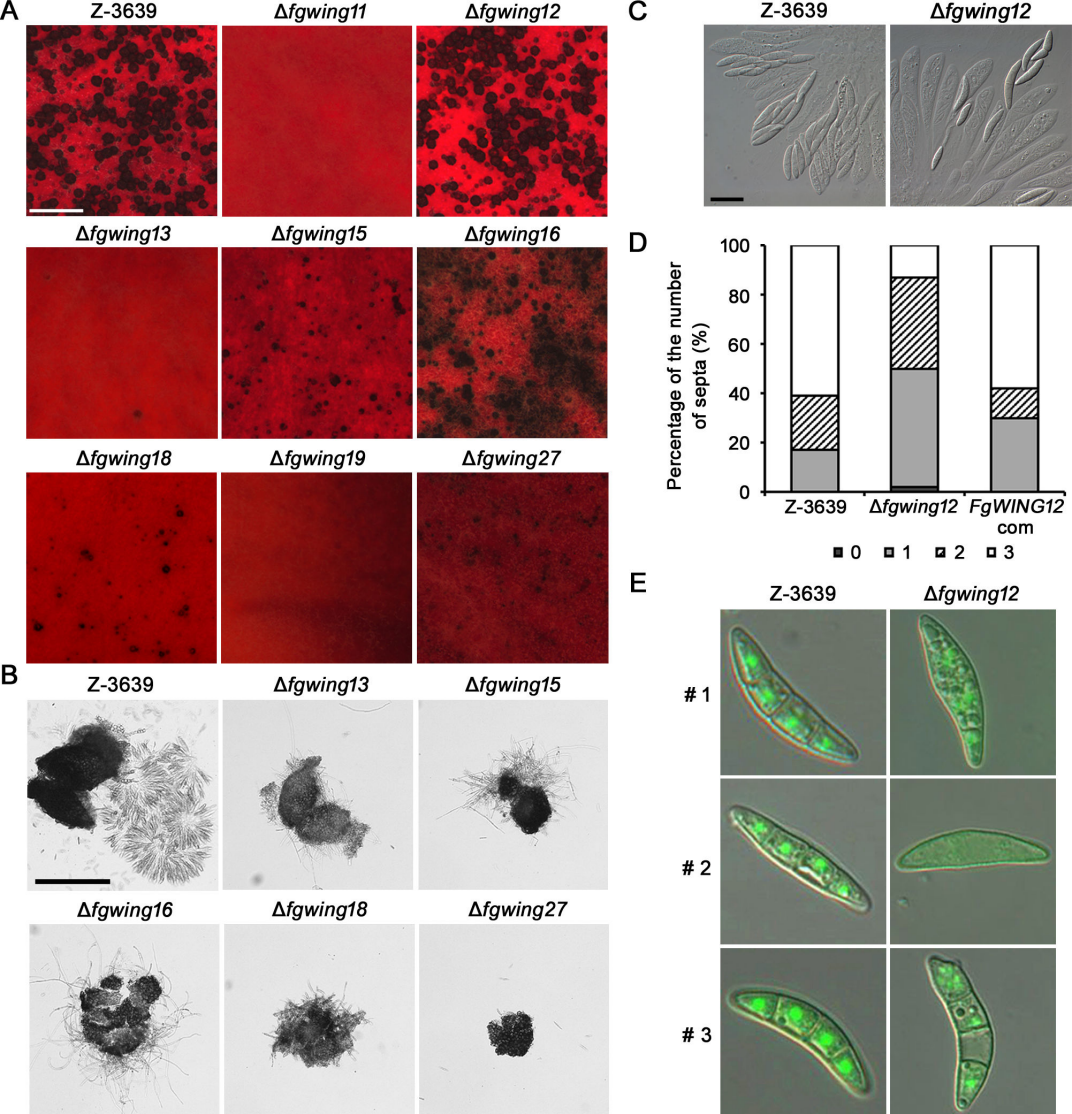
Sexual development of eight winged-helix DNA-binding protein mutants. (**A**) Perithecium formation on carrot agar media (CA). Photographs were taken 8 days after sexual induction. Scale bar, 1,000 µm. (**B**) Formation of asci rosettes. Perithecium was isolated from CA media and examined for ascus and ascospore development. Scale bar, 250 µm. (**C**) Asci and ascospores produced in the wild-type and ∆*fgwing12* deletion strains. Scale bar, 20 µm. (**D**) Percentage of ascospores with different numbers of septa. A total of 100 ascospores were observed per examination. (**E**) The representative ascospore images of the wild-type and ∆*fgwing12* deletion strains. The morphology of ascospores was observed 8 days after sexual induction.

To dissect the function of winged-helix DNA-binding proteins in fungal sexual reproduction, we generated deletion mutants of these genes by targeted gene replacement, and eight genes, except *FgWING4*, were successfully deleted (Fig. S1). We also generated complementary strains for all the deletion mutants. Upon examining the self-fertility of the *FgWING* deletion mutants, all eight mutants displayed defects in sexual reproduction as previously reported (Fig. 2A) (22). While the wild-type strain yielded perithecia 8 days after sexual induction, the Δ*fgwing11* and Δ*fgwing19* failed to produce any perithecia on carrot agar (CA) medium. Furthermore, in the Δ*fgwing13*, Δ*fgwing15*, Δ*fgwing18*, and Δ*fgwing27*, the number of initially formed perithecia was significantly reduced compared to the wild-type strain (Fig. 2A). Along with the Δ*fgwing16*, these strains produced small perithecia lacking internal maturation (Fig. 2B). The defects in sexual reproduction observed in these mutants were restored in the complemented strains, which produced normal perithecia (Fig. S2). The Δ*fgwing12* mutant produced normal perithecia but exhibited abnormal ascospore maturation. While the wild-type strain formed the usual eight-spored asci, the Δ*fgwing12* mutant showed partial development of spores within each ascus (Fig. 2C). Additionally, the formed ascospores had fewer septa compared to those in the wild-type strain (Fig. 2D). In the wild-type and complemented strains, 61% and 58% of ascospores possessed three septa, respectively. However, only 13% of Δ*fgwing12* ascospores had three septa, and 2% had no septa at all. We generated Δ*fgwing12* strains exhibiting green fluorescence in nuclei through an outcross between Δ*mat1-1*;hH1-GFP and Δ*fgwing12* mutants. Microscopic observations revealed that the deletion of *FgWING12* caused abnormal nuclear distribution and internal differentiation during ascosporogenesis (Fig. 2E). These results indicate that these eight winged-helix proteins play crucial roles in various stages of sexual reproduction.

### Eight winged-helix DNA-binding proteins are required for vegetative growth and/or asexual reproduction in *F. graminearum*

**Fig 3:**
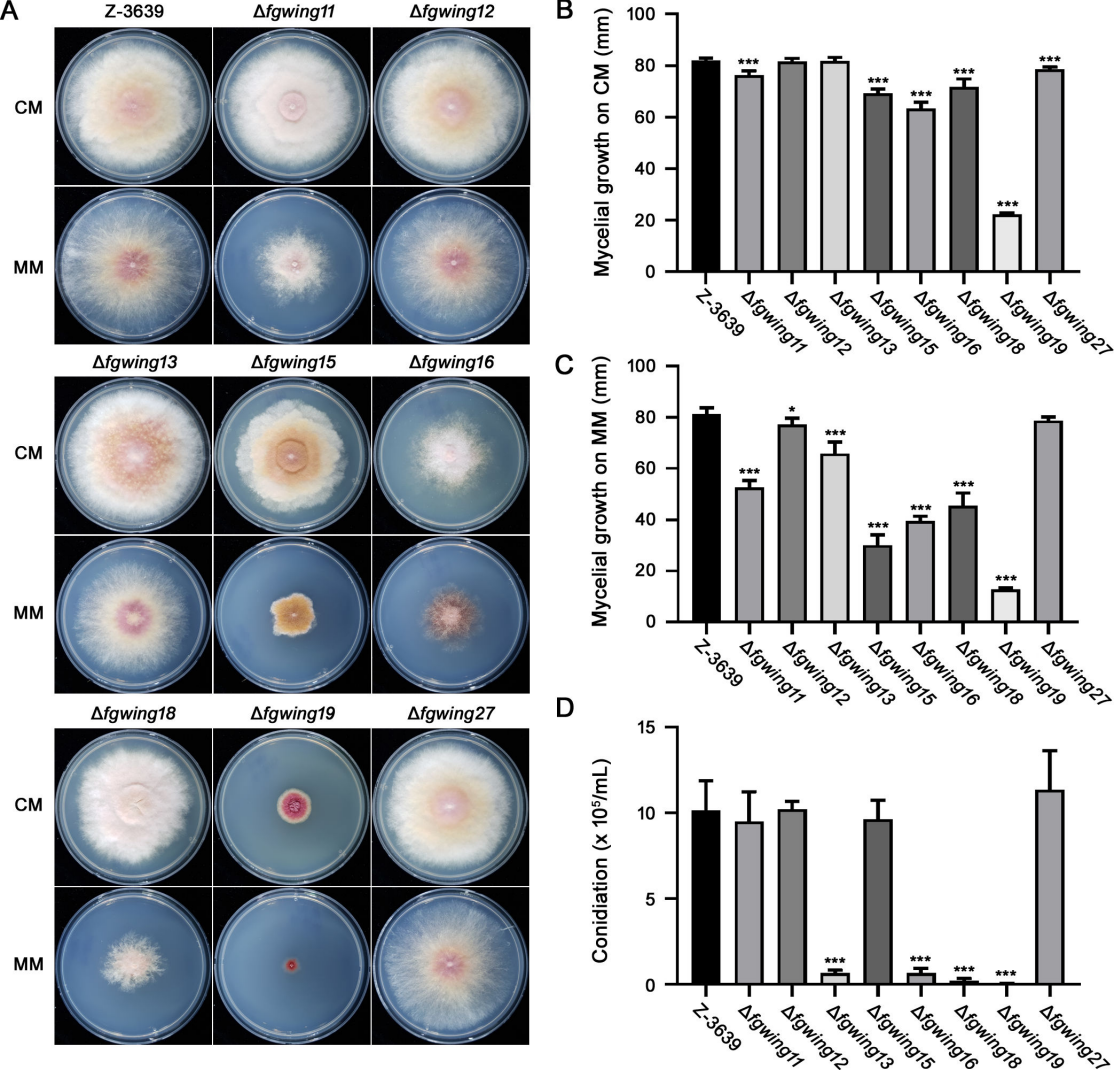
Vegetative growth and conidiation of eight winged-helix DNA-binding protein mutants. (**A**) Vegetative growth of each fungal strain on CM and MM. The fungal strains were inoculated on CM and MM, and pictures were taken 5 and 4 days after inoculation, respectively. (**B**) Colony diameters of each strain on CM. The diameter of the fungal colony was measured 5 days after inoculation. (**C**) Colony diameter of each strain on MM. The diameter of the fungal colony was measured 4 days after inoculation. (**D**) Conidiation of the wild-type and deletion mutants. Agar plugs of each strain were cultured in CMC media for 5 days, and the number of produced conidia was counted with a hemocytometer. Significant differences between the wild-type and deletion mutants were calculated using Student’s *t* test, and significance levels are indicated with asterisks (*P < 0.05, **P < 0.01, ***P < 0.001).

In addition to their role in sexual reproduction, we investigate the function of winged-helix DNA-binding proteins in fungal development. The vegetative growth of the eight mutants on complete medium (CM) and minimal medium (MM) was evaluated (Fig. 3A). While the Δ*fgwing12* and Δ*fgwing13* mutants exhibited normal growth, the other deletion mutants showed reduced growth rates on CM compared to the wild-type strain (Fig. 3B). Notably, the Δ*fgwing19* displayed a 73% decrease in growth compared to the wild-type strain (Fig. 3B). In MM, only the *FgWING27* deletion mutant maintained normal growth, with all other strains showing a significant decrease in growth compared to the wild-type strain (Fig. 3C). Furthermore, conidiation was examined for each strain, revealing severe defects in conidium production for Δ*fgwing13*, Δ*fgwing16*, Δ*fgwing18*, and Δ*fgwing19* mutants (Fig. 3D). These findings underscore the significant role of winged-helix DNA-binding proteins in vegetative growth and conidial production of *F. graminearum*, as well as in sexual reproduction.

### *FgWING27* is required for the stage-specific expression of *MAT* genes

**Fig 4:**
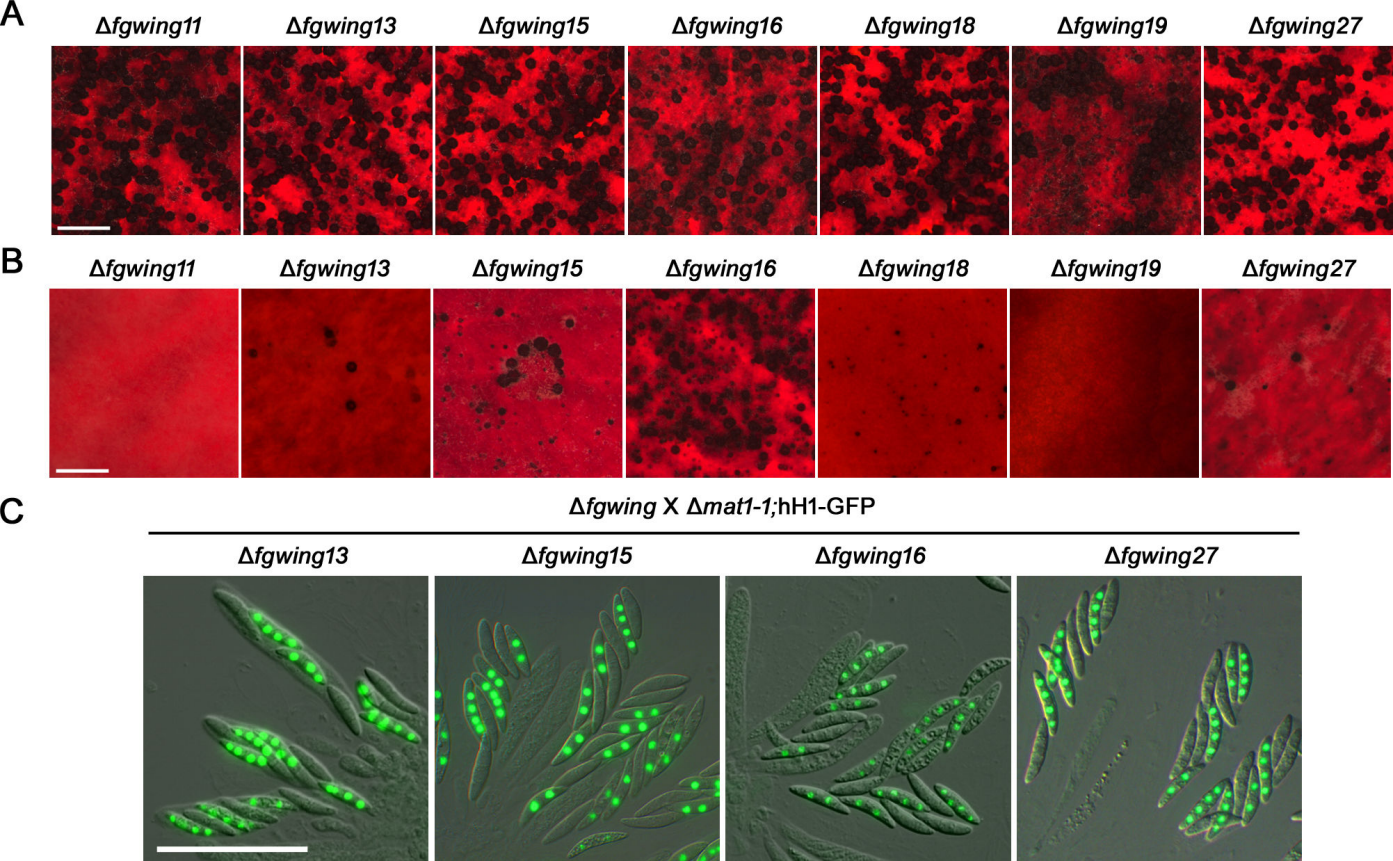
Male and female fertility of eight winged-helix DNA-binding protein mutants. (**A**) Male fertility of ∆*fgwing* mutants. Perithecium formation was examined from the outcross between ∆*mat1-1* hH1-GFP (♀) and ∆*fgwing* mutants (♂). Photographs were taken 8 days after outcrossing. Scale bar, 1,000 µm. (**B**) Female fertility of ∆*fgwing* mutants. Perithecium formation was examined from the outcross between ∆*fgwing* mutants (♀) and ∆*mat1-1*;hH1-GFP (♂). Scale bar, 1,000 µm. (**C**) Asci and ascospores produced by outcrossing between ∆*fgwing* mutants (♀) and ∆*mat1-1*;hH1-GFP (♂). The GFP signals in progeny were observed 8 days after outcross. Scale bar, 56.2 µm.

To further investigate the role of *FgWING* genes in sexual reproduction, we used the Δ*mat1-1* strain carrying hH1-GFP for outcrosses to see whether the *FgWING* mutants are male or female sterile (23). When the *FgWING* deletion mutants served as males (Δ*mat1-1;*hH1-GFP × Δ*fgwing*), there was a reduction in the number of perithecia formed in the outcross with Δ*fgwing19*. However, normal maturation of perithecia was achieved in outcrosses with all mutants, including Δ*fgwing19* (Fig. 4A). When *FgWING* mutants were outcrossed as females (Δ*fgwing* × Δ*mat1-1;*hH1-GFP), only four mutants—Δ*fgwing13*, Δ*fgwing15*, Δ*fgwing16*, and Δ*fgwing27*—retained female fertility (Fig. 4B). Although Δ*fgwing13*, Δ*fgwing15*, and Δ*fgwing27* formed a limited number of perithecia, the ratio of ascospores with or without the GFP signals was 1:1 in outcrosses with all four mutants, consistent with Mendelian segregation (Fig. 4C), suggesting that these genes are dispensable for female fertility.

**Fig 5:**
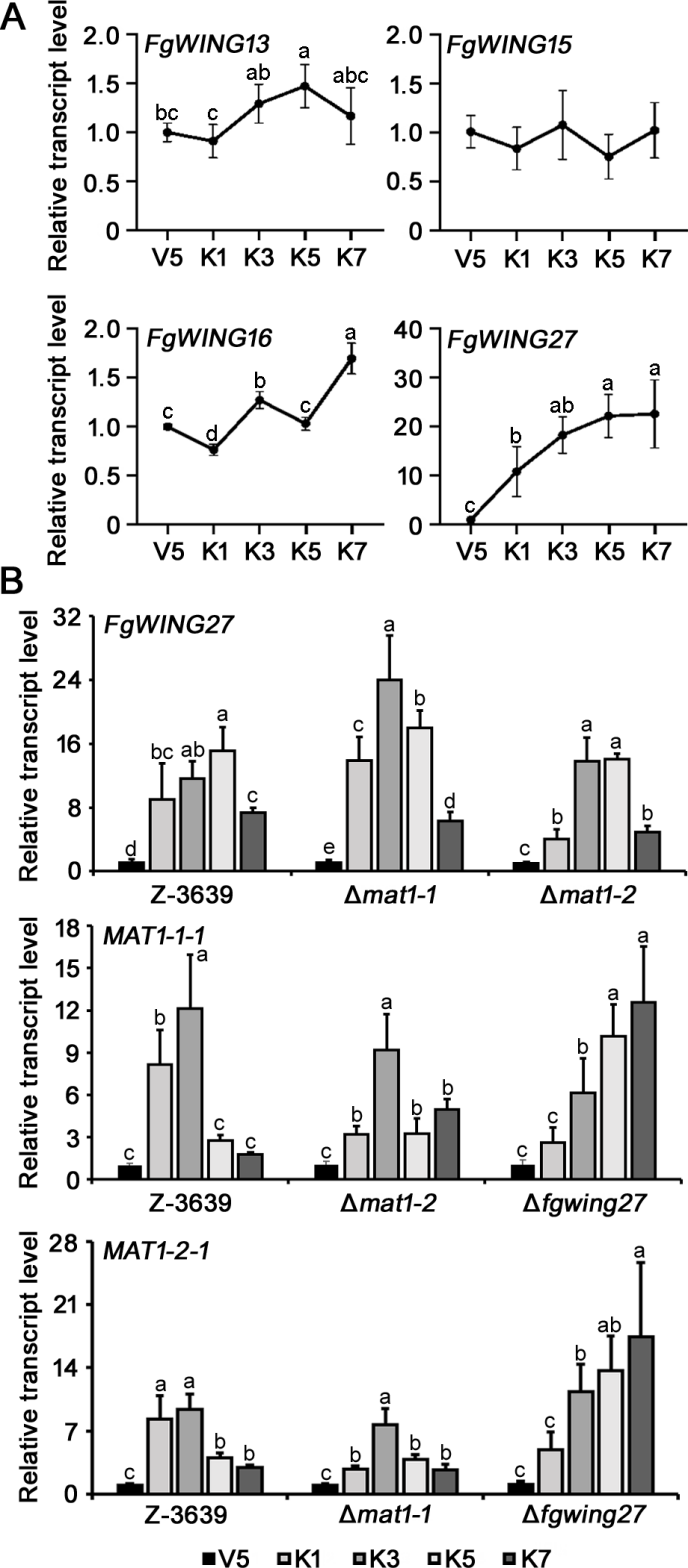
The transcriptional profiles of four *FgWING* and *MAT* genes during sexual reproduction. (**A**) The expression profiles of four *FgWING* genes during sexual reproduction. (**B**) Genetic relationship between *FgWING27* and *MAT* genes. The transcript levels of *FgWING27* and *MAT* genes were analyzed in the wild-type, ∆*mat1-1,* ∆*mat1-2*, and ∆*fgwing27* mutants at different sexual developmental stages. V5, vegetative growth stage for 5 days on carrot agar; K1, 1 day after sexual induction on carrot agar; K3, 3 days after sexual induction on carrot agar; K5, 5 days after sexual induction on carrot agar; K7, 7 days after sexual induction on carrot agar. Different letters indicate statistical differences determined by one-way ANOVA followed by Tukey’s *post hoc* test.

The characteristic of possessing both female and male fertility while being incapable of self-fertilization is a prominent trait of mating-type gene deletion mutants. To explore the potential role of these four genes as specific regulators in sexual reproduction, we analyzed their transcriptional profiles during sexual development (Fig. 5A). *FgWING13*, *FgWING15*, and *FgWING16* exhibited transcript levels that changed by less than twofold across sexual developmental stages compared to the vegetative growth stage, showing no significant differences in expression levels. Conversely, *FgWING27* expression was markedly induced at the early stage of sexual development, displaying over a 10-fold increase compared with the vegetative growth stage.

To examine the genetic relationship between *MAT* genes and *FgWING27*, we analyzed the transcriptional changes of *FgWING27* in *MAT1-1* and *MAT1-2* deletion mutants. In the wild-type strain, the transcript level of *FgWING27* drastically increased on the first day after induction, peaked, and then decreased after 5 days of sexual induction (Fig. 5B). Interestingly, the transcriptional profile of *FgWING27* in the *mat1-1* mutant was similar to that of the wild type. In the *mat1-2* mutant, *FgWING27* transcript levels were diminished on the first day after induction of sexual reproduction. However, it still exhibited a peak expression pattern between the third and fifth days, with no significant differences compared to those in the wild-type and *mat1-1* strains. These findings prompted us to examine the expression patterns of *MAT1-1-1* and *MAT1-2-1* in the *fgwing27* mutant, revealing notable changes in their expression patterns following *FgWING27* deletion. In the wild-type strain, both *MAT1-1-1* and *MAT1-2-1* were highly induced during early sexual development, declining after 5 days of sexual induction. In the deletion mutant of other *MAT* genes, slight differences in transcript levels were observed, but their expression patterns were similar to those of the wild-type strain. However, in the absence of *FgWING27*, late-stage transcriptional inhibition of both *MAT* genes did not occur, and their transcript levels gradually increased until the late stages of sexual development. These findings underscore the crucial role of Fgwing27 in the transcriptional regulation of mating-type genes during late-stage sexual development.

### Fgwing27 acts as a positive regulator of a gene related to sexual reproduction

**Fig 6:**
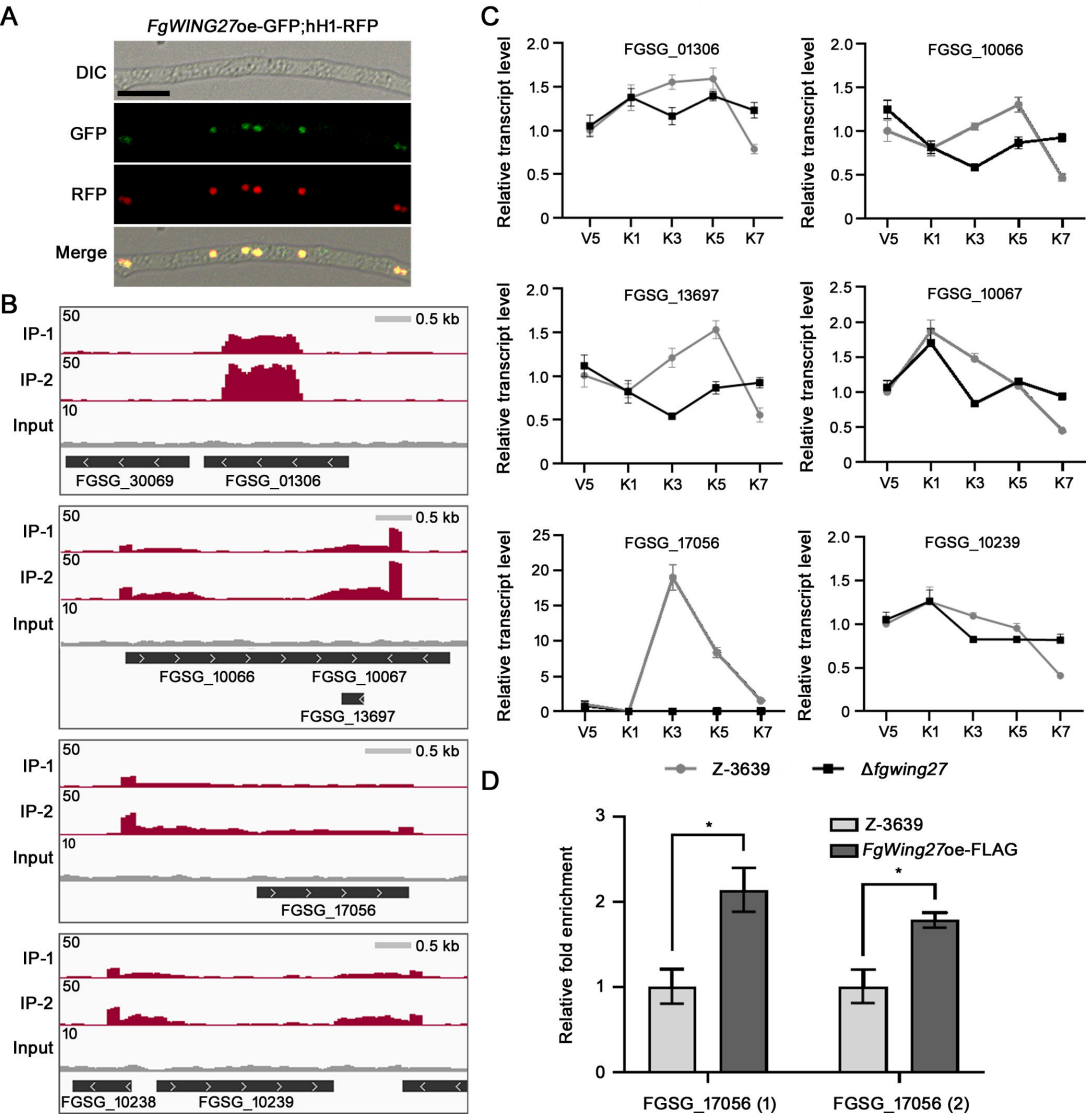
Identification of Fgwing27 downstream genes. (**A**) Subcellular localization of Fgwing27 in mycelia of *F. graminearum*. Scale bar, 14 µm. (**B**) Visualization of the ChIP-seq peaks using an integrative genome viewer (IGV). (**C**) The transcriptional profiles of putative Fgwing27 downstream genes during sexual reproduction. V5, vegetative growth stage for 5 days on carrot agar; K1, 1 day after sexual induction on carrot agar; K3, 3 days after sexual induction on carrot agar; K5, 5 days after sexual induction on carrot agar; K7, 7 days after sexual induction on carrot agar. (**D**) ChIP-qPCR analysis. *UBH1* was used as an internal control. Asterisks represent significant differences (*P < 0.05; *t* test).

To determine whether Fgwing27 functions by directly binding to genes essential for sexual reproduction, we generated strains overexpressing *FgWING27* fused with GFP or FLAG, which enabled Fgwing27 to be expressed under normal conditions. For confirmation of the subcellular localization of Fgwing27, the *FgWING27*oe-GFP;hH1-RFP strain was generated by outcrosses between *FgWING27*oe-GFP and the Δ*mat1-1* mutant strain carrying red fluorescent protein (RFP) fused to histone H1 protein. The fluorescence signal of *FgWING27*oe-GFP colocalized with that of hH1-RFP, indicating that Fgwing27 is localized to nuclei (Fig. 6A).

We then conducted chromatin immunoprecipitation followed by sequencing (ChIP-seq) using the FLAG-tagged Fgwing27 strain to identify the downstream targets of Fgwing27. Across two independent experiments, we identified 45 peak-associated genes, and the top five enriched regions were visualized using an integrative genome viewer (Fig. 6B). Notably, these peaks exhibited symmetrical patterns over a broad range. This suggests a potential bispecific recognition or involvement of Fgwing27 in inducing DNA structural changes, thereby raising the possibility of regulating gene expression by modulating the spatial positioning of the genes. We further examined the transcriptional levels of eight genes adjacent to the top five peak-enriched regions during sexual development in both the wild-type and Δ*fgwing27* strains to elucidate the regulatory role of Fgwing27. Except for two genes, *FGSG_10238* and *FGSG_ 30069*, which showed undetectable transcript levels, the remaining six genes exhibited distinct transcriptional patterns during sexual development upon *FgWING27* deletion. Among them, *FGSG_17056* showed a significant 19-fold upregulation at 3 days post-sexual induction compared to the vegetative growth stage in the wild-type strain; however, no change in expression was observed in the Δ*fgwing27* strain (Fig. 6C). To validate the direct regulation of Fgwing27 on *FGSG_17056*, we performed chromatin immunoprecipitation followed by quantitative PCR (ChIP-qPCR), and found a significant enrichment of *FGSG_17056* in the immunoprecipitated *FgWING27*oe-FLAG sample (Fig. 6D).

### *Fg17056*, a downstream gene of Fgwing27, is essential for sexual reproduction

**Fig 7:**
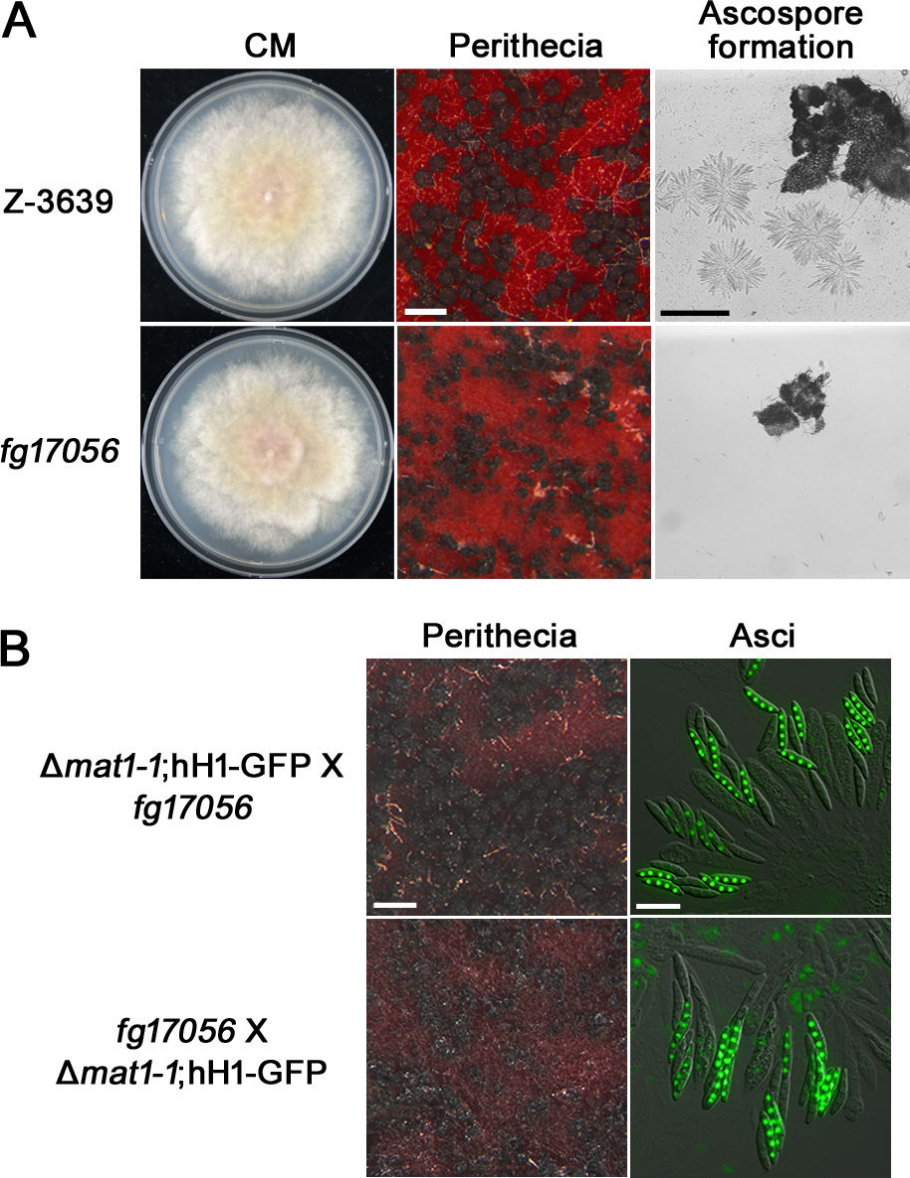
Sexual reproduction of *fg17056* mutant. (**A**) Vegetative growth and sexual development of wild-type and *fg17056* strains. Mycelial growth on CM was observed 5 days after inoculation, and perithecia and ascospore formation were examined 8 days after sexual induction. White scale bar, 500 µm; black scale bar, 250 µm. (**B**) Male and female fertility of *fg17056*. The *fg17056* strain, as either a male or female strain, was outcrossed with ∆*mat1-1*; hH1-GFP strain, and photos were taken 8 days after outcrossing. Scale bar in “Perithecia” panel, 500 µm; in “Asci” panel, 20 µm.

To investigate whether the function of Fgwing27 on sexual reproduction governs through the direct regulation of the downstream gene, we dissected the role of *FGSG_17056* in sexual reproduction. We generated deletion mutants for *FGSG_17056*, designated *fg17056*, and assessed their phenotypic traits related to vegetative growth and sexual reproduction. The *fg17056* mutants exhibited normal vegetative growth but abnormal sexual development (Fig. 7A). Notably, in the *fg17056* mutants, only small and immature perithecia were produced without ascospore formation. The male and female fertility of *fg17056* was also examined using the Δ*mat1-1*;hH1-GFP strain for outcrosses. It was revealed that *fg17056* mutants retain both male and female fertility, which is identical to the phenotypes of Δ*fgwing27* mutants (Fig. 7B). These findings demonstrate that the function of Fgwing27 on sexual reproduction is mediated by the direct regulation of *Fg17056* in *F. graminearum*.

## DISCUSSION

The sexual developmental process of fungi is intricately regulated by a complex genetic regulatory network, and numerous studies have been conducted to elucidate this intricate process. Transcriptomic approaches on the *MAT* genes have revealed their role as master regulators of sexual development and have identified downstream genes required for sexual development in fungi. However, the complex nature of sexual reproduction processes requires further comprehensive investigation. Additionally, the functional requirements of individual *MAT* genes in sexual reproduction vary among fungal species (11, 24), highlighting the need for species-specific studies on this biological process. In this context, we focused on the winged-helix DNA-binding protein implicated in sexual reproduction, uncovering the pivotal role of Fgwing27, which is highly conserved within the *Fusarium* genus, as a transcriptional regulator during sexual reproduction.

In fungal species, the roles of winged-helix DNA-binding proteins have been extensively studied, with a primary focus on two families: the forkhead-box family and the RFX family. The forkhead box family was named after the two spiked-head structures in embryos observed in the mutant of *Drosophila melanogaster* (25), and their roles in the regulation of cell cycle and cellular differentiation have been extensively investigated from yeast to animals (19, 26). In *Saccharomyces cerevisiae*, two forkhead transcription factors, Fkh1 and Fkh2, have been identified as regulators of cell-cycle genes (27, 28). Forkhead transcription factors also play critical roles in asexual and sexual development, which encompass complex structural changes. Forkhead transcription factors are indispensable for growth and reproduction in several fungal species, including *Aspergillus nidulans*, *Sclerotinia sclerotiorum*, and *Magnaporthe oryzae* (29–31). RFX family members are also conserved in animals, fungi, and amoebozoa (32). Although RFX transcription factors are well-known for their key role in regulating ciliogenesis in the animal kingdom (20, 32), in fungi where cilia are absent, they have been reported to be involved in fungal morphogenesis and genome integrity maintenance (33). For instance, in *Acremonium chrysogenum*, the RFX transcription factor Cpcr1 governs morphological development and is required for hyphal fragmentation (34). Similarly, in *Penicillium marneffei*, the RFX protein RfxA plays a pivotal role in morphogenesis and fungal growth (35). As previously reported for the functions of forkhead and RFX transcription factors, many winged-helix DNA-binding proteins in *F. graminearum* play a crucial role in fungal development and sexual reproduction. Fgwing4, for which gene deletion was unsuccessful, is an ortholog of the *S. cerevisiae* Cdc6, which is necessary for the formation of pre-replicative complex (36, 37). Cdc6 is crucial for the initiation of DNA replication, and studies using conditional expression have shown its necessity for cell viability (38, 39). Fgwing12 is an ortholog of Rec12/Spo11, and Rec12 is necessary for the initiation of meiotic recombination by forming DNA double-strand breaks (40, 41). During meiosis, Rec12 is required for pairing the chromosomal arm regions and stabilizing chromosomes (42). In *Neurospora crassa*, the deletion of *SPO11* caused defects similar to those observed in the Δ*fgwing12* mutant, resulting in abnormal chromosome segregation and sporogenesis (43), which support the role of Fgwing12 in ascosporogenesis as an ortholog of Rec12/Spo11 in *F. graminearum*. Also, Fgwing16, which exhibited defects in perithecia formation upon deletion, has been studied as Rfx1 and was found to play an essential role in maintaining genome integrity by regulating the DNA repair system (44). These findings emphasize that winged-helix DNA-binding proteins are involved in cell cycle regulation and DNA integrity, having broad significance in cell differentiation and development.

In this study, we revealed that four self-sterile mutants, Δ*fgwing13*, Δ*fgwing15*, Δ*fgwing16*, and Δ*fgwing27*, retained both female and male fertilities. This indicates that while the deletion of these genes abolishes selfing ability, the capacity for outcrossing remains preserved, which is an identical trait to *MAT* idiomorphs (23). This suggests that these genes may play pivotal roles in the reproductive strategy of the fungus or act as integral components alongside *MAT* genes. Notably, Fgwing27 is a protein unique to the *Fusarium* genus, exhibiting defects solely in sexual reproduction upon deletion. A previous study positioned *FgWING27* as a downstream gene of the *MAT* genes, with reduced transcript levels in *MAT*-deletion mutants 2 days after sexual induction (13). Similarly, in our study, we observed a slight decrease in the expression of *FgWING27* during the early stages of sexual development (K1) upon deletion of the *MAT1-2*. However, the time-course expression profiles of *FgWING27* and *MAT* genes during sexual reproduction revealed that the deletion of *FgWING27* resulted in aberrant expression of *MAT* genes, characterized by a gradual increase throughout sexual reproduction. These results indicate that Fgwing27 functions as a regulatory factor for *MAT* gene expression in the late stages of sexual reproduction. In *F. graminearum*, several studies consistently have reported a reduction of *MAT* genes expression in the late-stage of sexual reproduction (24, 45, 46), but the underlying mechanisms have been scarcely studied. Based on the phenotype of *MAT* gene overexpression mutants, the reduction in transcript levels of *MAT* genes is dispensable for the sexual reproduction, as these mutants successfully undergo sexual differentiation (13). However, in *A. nidulans*, it was reported that the overexpression of *MAT* genes caused suppression of vegetative growth and stimulation of sexual differentiation under unfavorable conditions for sexual reproduction (47). Also, in *Penicillium chrysogenum*, overexpression of *MAT1-2-1* affects the spore germination (48). These findings suggest that stage-specific regulation of *MAT* genes is required to maintain a balance between vegetative growth, asexual and sexual reproduction.

In addition to the role of *FgWING27* in the stage-specific genetic regulation of *MAT* genes, we further identified *Fg17056* as a downstream gene of Fgwing27 using ChIP-seq analysis. Although the mode of binding of Fgwing27 on DNA is not yet clear, Fgwing27 directly regulates the expression of *Fg17056*. The phenotype of the *fg17056* deletion mutant, which exhibits self-sterility while retaining both male and female fertility, is identical to the defects caused by the deletion of Fgwing27. This strongly supports that the involvement of Fgwing27 in sexual reproduction is mediated through *Fg17056*. The gene *Fg17056* encodes n-alkane-inducible cytochrome P450 belonging to the CYP52 family. A previous study on cytochrome P450 monooxygenases in *F. graminearum* revealed that *Fg17056* is required for determining perithecia number and perithecium maturation during sexual development (49). The CYP52 family hydroxylates n-alkanes to fatty alcohols, which are further metabolized via β-oxidation and used for synthesizing membrane or storage lipids (50, 51). Lipid metabolism is crucial for fungal sexual reproduction as it generates and utilizes energy sources (52), and lipid biosynthesis occurs at the early stages of sexual reproduction, with storage lipids being used for perithecium formation (46, 53, 54). *Fg17056* has also been identified as a gene downstream of Gpa1, a G protein alpha subunit 1 (55). The G protein complex is crucial for pheromone signaling, triggering the activation of the mitogen-activated protein kinase signal transduction pathway, and the alpha subunit of the G protein is essential for sexual reproduction (56). These findings suggest that *Fg17056* plays a significant role both functionally and within the genetic regulatory network related to sexual reproduction.

In conclusion, we confirmed the role of winged-helix DNA-binding proteins in the sexual reproduction of *F. graminearum*, with a particular focus on the Fgwing27. We revealed the pivotal role of Fgwing27 in controlling the expression of *MAT* genes during the late stages of sexual development and elucidated the mechanisms by which Fgwing27 modulates the expression of a key gene involved in sexual reproduction using ChIP-seq analysis. Our study offers new perspectives on fungal sexual reproduction by highlighting the importance of stage-specific regulatory factors in sexual reproduction.

## MATERIALS AND METHODS

### Fungal strains and media

The *F. graminearum* wild-type strain Z-3639 (57) and transgenic strains derived from this strain were maintained in 20% glycerol at −80°C. All culture media used in this study were prepared and used according to “The *Fusarium* Laboratory Manual” (58).

### DNA and RNA extraction

Fungal genomic DNA was extracted using the CTAB procedure followed as previously described (58). Total RNA was isolated using the Easy-Spin Total RNA Extraction Kit (Intron Biotech, Seongnam, Republic of Korea).

### Nucleic acid manipulations and fungal transformation

To generate the constructs for targeted gene deletion, the double joint (DJ) PCR method was used (59). Briefly, the 5′ and 3′ flanking regions of the target open reading frame (ORF) were amplified with the primer pairs 5F-5R and 3F-3R, respectively, and fused with the geneticin resistance cassette amplified from pII99 (60) by overlapping PCR. Using nested primers, the final constructs were amplified to split the marker genes, and the resulting PCR amplicons were used for fungal transformation.

The yeast gap repair approach was used to generate *FgWING* complementation strains (61). The ORF and promoter regions of each gene were amplified and co-transformed with the *Xho*I-digested pDL2 vector into yeast strain PJ69-4A. The recombinant plasmid was isolated from yeast transformants and subsequently transformed into *Escherichia coli* DH10B. The resulting plasmids were verified using sequencing analysis. To generate the GFP-/FLAG-tagged Fgwing27 overexpression strains, the ORF of Fgwing27 was amplified. Co-transformation was conducted with *XhoI*-digested pDL2 or pHZ126 vectors, respectively, and recombinant plasmids were obtained as described above.

All generated DNA fragments or recombinant plasmids were transformed into fungal protoplasts via PEG-mediated transformation, as previously described (62). The PCR primers used in this study were synthesized by an oligonucleotide synthesis facility (Bioneer, Daejeon, Republic of Korea) and are listed in Table S2.

### Sexual development assay and microscopic observation

For induction of sexual reproduction, each fungal strain was inoculated on CA medium, and after 5 days of incubation, aerial mycelia were removed from the media with 400 µL of 2.5% tween 60 solutions. For outcrossing, a female strain grown on CA was fertilized with 1 mL of conidial suspension from a male strain. The resulting media were further incubated under near-UV light (wavelength: 352 nm; Sankyo Denki, Tokyo, Japan) and perithecia formation was observed 7 days after sexual induction or fertilization using a SteREO Lumar V12 (Carl Zeiss, Oberkochen, Germany). For ascospore discharge, a semi-circular agar block covered with mature perithecia was cut and placed on a glass slide. After incubation for 24 h, ejected ascospores were observed.

To observe the GFP signals in the progeny, the perithecia were placed on glass slides and squeezed with a cover slide. The fluorescence signals of the ascospores were observed using a DM6 B microscope (Leica Microsystems) equipped with a Leica DMC6200 camera.

### qRT-PCR

Total RNA was extracted from the mycelia and perithecia samples harvested at different stages, and first-strand cDNA was synthesized using the SuperScript III First-Strand Synthesis System (Invitrogen, Carlsbad, CA, USA) following the manufacturer’s protocol. qRT-PCR was performed using the CFX Real-Time PCR System (Bio-Rad, Hercules, CA, USA) with iTaq Universal SYBR Green Supermix (Bio-Rad). Relative transcript levels were calculated through the 2^−ΔΔCT^ method (63). For normalization, the endogenous housekeeping gene ubiquitin C-terminal hydrolase (*UBH1*) was used as an internal control. The primers used for qRT-PCR are listed in Table S2.

### ChIP-seq analysis and ChIP-qPCR

ChIP samples were prepared for sequencing as previously described (64), and sequencing was performed using the Illumina NovaSeq 6000 platform (Illumina, San Diego, CA, USA). Sequencing data were analyzed using the Galaxy web-based platform. After trimming with Trim Galore (version 0.6.7), all sequence reads were mapped to the *F. graminearum* PH-1 genome using Bowtie2 (version 2.2.7.1) (65). The mapped read enrichment was normalized using the bamCoverage tool (version 3.5.1.0.0) (66) and visualized using the Integrative Genomics Viewer. All ChIP-seq data have been deposited in the NCBI Sequence Read Archive under the accession number PRJNA1099902.

For ChIP-qPCR, primers were designed to be located in the peak regions. The relative enrichment level was determined using the 2^−ΔΔCT^ method (63), and *UBH1* was used as an internal control. The primers used in this study are listed in Table S2.

## Data Availability

All ChIP-seq data have been deposited in the NCBI Sequence Read Archive under the accession number PRJNA1099902.

## SUPPLEMENTAL MATERIAL

Supplemental material msphere.00511-24-s0001.pdf Tables S1 and S2; Fig. S1.

ASM does not own the copyrights to Supplemental Material that may be linked to, or accessed through, an article. The authors have granted ASM a non-exclusive, world-wide license to publish the Supplemental Material files. Please contact the corresponding author directly for reuse.
