# Supplementary material for: A winged-helix DNA-binding protein is essential for self-fertility during sexual development of the homothallic fungus Fusarium graminearum
Source: mSphere. 2024 Aug 27;9(9):e00511-24. doi: 10.1128/msphere.00511-24 (PMC11423578; doi:10.1128/msphere.00511-24)
Supplement: Supplemental material — Tables S1 and S2; Fig. S1. [file msphere.00511-24-s0001.pdf]

Supplemental materials

A winged helix DNA-binding protein is essential for self-fertility during sexual development of the homothallic fungus *Fusarium graminearum*

Jiyeun Park[1#], Hosung Jeon[1#], Aram Hwangbo[1], Kyunghun Min[2], Jaeho Ko[1], Jung-Eun Kim[3], Sieun Kim[4], Ji Young Shin[5], Yong-Hwan Lee[1,6,7,8,9,10], Yin-Won Lee[1], and Hokyoung Son[1,6]

[1]Department of Agricultural Biotechnology, Seoul National University, Seoul 08826, Republic of Korea

[2]Department of Plant Science, Gangneung-Wonju National University, Gangneung 25457, Republic of Korea

[3]Research Institute of Climate Change and Agriculture, National Institute of Horticultural and Herbal Science, Jeju 63240, Republic of Korea

[4]Horticultural and Herbal Crop Environment Division, National Institute of Horticultural and Herbal Science, Wanju 55365, Republic of Korea.

[5]Honam National Institute of Biological Resources, Mokpo 58762, Republic of Korea

[6]Research Institute of Agriculture and Life Sciences, Seoul National University, Seoul 08826, Republic of Korea

[7]Interdisciplinary Programs in Agricultural Genomics, Seoul National University, Seoul 08826, Republic of Korea

[8]Center for Plant Microbiome Research, Seoul National University, Seoul 08826, Republic of Korea

[9]Plant Immunity Research Center, Seoul National University, Seoul 08826, Republic of Korea

[10]Plant Genomics and Breeding Institute, Seoul National University, Seoul 08826, Republic

of Korea

[#]Jiyeun Park and Hosung Jeon contributed equally to this work.

Address correspondence to Hokyoung Son, hogongi7@snu.ac.kr

Table S1. Nine winged helix-like DNA binding proteins implicated in sexual reproduction

Protein name	Locus ID	Description	Reference
Fgwing4	FGSG_01030	cell division control 6	(1)
Fgwing11	FGSG_05520	ubiquitin ligase subunit	(1)
Fgwing12	FGSG_05949	meiotic recombination rec12	(1)
Fgwing13	FGSG_06228, FGSG_16620	developmental regulator flbA	(1)
Fgwing15	FGSG_06944	HCM1-transcription factor	(1)
Fgwing16	FGSG_07420	cephalosporin c regulator	(1, 2)
Fgwing18	FGSG_08481	histone acetyltransferase	(1)
Fgwing19	FGSG_08572	Citron partial	(1)
Fgwing27	FGSG_11826, FGSG_15782	forkhead box o4	(1)

Table S2. Primers used in this study

Primer name	Sequence (5' to 3')	Description
FgWING11_5F	GATCTTTGAGTTACCGCGTCCTACAG	For the construction of *FgWING11* deletion mutant
FgWING11_5N	GCGATCTCTGACTGCGGCTGAT	
FgWING11_5R	gcacaggtacacttgtttagagCAGAGGGCTGCTGCGTCAAGT	
FgWING11_3F	ccttcaatatcatcttctgtcgAGCTCAAAGAGAGAAAGGCCAAAGAT	
FgWING11_3N	CGTGGTTTTGCGGCGTAGG	
FgWING11_3R	CTTCCTTGTCCGCTGGTGTTTTATG	
FgWING12_5F	TGGCCTTCATTGCTTCGGAGATAC	For the construction of *FgWING12* deletion mutant
FgWING12_5N	AATGTCGTTGCGGTGGCTTGTC	
FgWING12_5R	gcacaggtacacttgtttagagGCTTGAGCAATGTATGGGTCTTGG	
FgWING12_3F	ccttcaatatcatcttctgtcgATTGGCGAAGCATTGATCAGTGA	
FgWING12_3N	CGCGGATCTTTCGACTTTTTACTG	
FgWING12_3R	CCTGTCAAGATTGAAGGCGATTACC	
FgWING13_5F	CAGGTGTTTAAAGGGACGGTTGG	For the construction of *FgWING13* deletion mutant
FgWING13_5N	TAGTCGTCATACAGCGCCTCAAAA	
FgWING13_5R	gcacaggtacacttgtttagagGCAAAGGCGAGAATAGGTAAGGTA	
FgWING13_3F	ccttcaatatcatcttctgtcgGCTCGAAAGGAAAGCTCAAGTCTA	
FgWING13_3N	TGCTTTGATGACATGTCTTGGTGAC	
FgWING13_3R	AACGAAGAGCATTGTTTGGTTGTTC	
FgWING15_5F	AGTTTCTCCTTCGACCCACACCTC	For the construction of *FgWING15* deletion mutant
FgWING15_5N	CATCAGTCTCGTCTCGCTTTGTCC	
FgWING15_5R	gcacaggtacacttgtttagagAAGTGGAAGGGGATGACGGTAA	
FgWING15_3F	ccttcaatatcatcttctgtcgACGTGGCAAGACGACGAAATAA	
FgWING15_3N	GCCTTTGAGCGACAGATAGACACC	
FgWING15_3R	TGGCTCAGTGACGTTGTTTAGGAG	
FgWING16_5F	CCAATACGTAAGAGGAGCACAGTT	For the construction of *FgWING16* deletion mutant
FgWING16_5N	GTGGCTTGTGACCCTGTGGACT	
FgWING16_5R	gcacaggtacacttgtttagagCGGGGACGTTGTTGATTTAGG	
FgWING16_3F	ccttcaatatcatcttctgtcgCTCAGAACCAAGGAGAACGAGCAC	
FgWING16_3N	GCTTTGCTGCTGCTGATGGTGA	
FgWING16_3R	CTTCTCGCCAAAGCTGATGACAC	
FgWING18_5F	TGAGACAGAGATTACAACCAACGGAAC	For the construction of *FgWING18* deletion mutant
FgWING18_5N	GCCCATTCTACAGGCCTTGCTATTT	
FgWING18_5R	gcacaggtacacttgtttagagATTGTGGGGGTGCCGTGTATT	
FgWING18_3F	ccttcaatatcatcttctgtcgGACCCAAGAGCTCATCAAAGAAAAAG	
FgWING18_3N	TGGAAGAGGGTACAAATATCGTTGAGG	
FgWING18_3R	AGCAGACCTAAAGCTTTAGCACAGAAGT	
FgWING19_5F	CAGCAGTAGAACCGGACATTAGGG	For the construction of *FgWING19* deletion mutant
FgWING19_5N	AAGGATGCCCCCAGTGTCAGAA	
FgWING19_5R	gcacaggtacacttgtttagagGGTTTAAGGGGAGACGTTTGATGA	
FgWING19_3F	ccttcaatatcatcttctgtcgATTTTGTTTGCACACGAAGACGAC	
FgWING19_3N	GACCGACGAGAATAAATGAGCCCAC	
FgWING19_3R	AAATGATTGTCGTGGTTAGCAAAGG	
FgWING27_5F	ATCAAACCATGCGTCAATCAGTGT	For the construction of *FgWING27* deletion mutant
FgWING27_5N	GCAGTGACAACGACTATATGCTCCAACAA	
FgWING27_5R	gcacaggtacacttgtttagagTGTGTTGAAGTCAAGGACGGTGTT	
FgWING27_3F	ccttcaatatcatcttctgtcgACCAGTCAAACAACCCCTCCTC	
FgWING27_3N	TGGACAACTTTCAAACGATGGATG	
FgWING27_3R	GTCTGGGATGAATAGGATGATGGG	

FgWING11_GFP_F	tatagggcgaattgggtactcaaattggttCGACGAGAGACTGATCCAGAGGTAG	For amplification of the native promoter and ORF regions of *FgWING11*
FgWING11_GFP_R	cccggtgaacagctcctcgcccttgctcacAGCTAGGTACACATATGCATTGCCCT	
FgWING12_GFP_F	tatagggcgaattgggtactcaaattggttCAACAAGACGAAGGGCAACATAGTT	For amplification of the native promoter and ORF regions of *FgWING12*
FgWING12_GFP_R	cccggtgaacagctcctcgcccttgctcacAATCATATCACTGATCAATGCTTCGCCAA	
FgWING13_GFP_F	tatagggcgaattgggtactcaaattggttAATGCCTTGTTCTTCTTTGACCC	For amplification of the native promoter and ORF regions of *FgWING13*
FgWING13_GFP_R	cccggtgaacagctcctcgcccttgctcacCTTTCGGTTGGACCTGCTTTG	
FgWING15_GFP_F	tatagggcgaattgggtactcaaattggttAGTACTTACTACAGGCCATTGATACAGATG	For amplification of the native promoter and ORF regions of *FgWING15*
FgWING15_GFP_R	cccggtgaacagctcctcgcccttgctcacAAATTGTGTTGAAAAACTTCGGGGTAAT	
FgWING16_GFP_F	tatagggcgaattgggtactcaaattggttCAACATGCTTTAGGGTGAGTGACG	For amplification of the native promoter and ORF regions of *FgWING16*
FgWING16_GFP_R	cccggtgaacagctcctcgcccttgctcacTAGCCCGCCTGGGGCGAACT	
FgWING18_GFP_F	tatagggcgaattgggtactcaaattggttAGGGTGCCTCTGCCTTGTCTGT	For amplification of the native promoter and ORF regions of *FgWING18*
FgWING18_GFP_R	cccggtgaacagctcctcgcccttgctcacAATGTCCTCCCCTTCGGCATCA	
FgWING19_GFP_F	tatagggcgaattgggtactcaaattggttGTACACATGCATGACGAGGACAGAG	For amplification of the native promoter and ORF regions of *FgWING19*
FgWING19_GFP_R	cccggtgaacagctcctcgcccttgctcacTGAACCAAGCAGTTCTGACTTGCG	
FgWING27_GFP_F	tatagggcgaattgggtactcaaattggttGTCCAACCAACATCGGCACATT	For amplification of the native promoter and ORF regions of *FgWING27*
FgWING27_GFP_R	cccggtgaacagctcctcgcccttgctcacGAGTCCCTGGTTGTTCCAATTGTAG	
FgWING13 qRT-F	TTCGAAGCTCATCAAACCAATCACT	For qRT-PCR of FgWING13
FgWING13 qRT-R	CTGTGGTTGTGTAAGGCTGAGACG	
FgWING15 qRT-F	ATAAAATCCGAGCCAATGACTGCG	For qRT-PCR of FgWING15
FgWING15 qRT-R	CATGGCAATCAAGTCGGCGTAAC	
FgWING16 qRT-F	CGGAGAAATCAACACAGGCAACAG	For qRT-PCR of FgWING16
FgWING16 qRT-R	TGGGCGAAGCGTAAGGGAATC	
FgWING27 qRT-F	GAATCAGCAACAGCGGTCACATC	For qRT-PCR of FgWING27
FgWING27 qRT-R	CCACTGCTTGGGGAACTTTGATA	
MAT1 qRT-F	CTGGAAGAACTGGGCATCGTAA	For qRT-PCR of *MAT1-1-1*
MAT1 qRT-R	GATATTCTTGTGGCTGGCTACTTT	
MAT2 qRT-F	TGGCAGACGACATTAAGGAGGAGCAC	For qRT-PCR of *MAT1-2-1*
MAT2 qRT-R	TGAGCAGCGACAGCAGCAGCAAGAA	
UBH1 qRT-F	GTTCTCGAGGCCAGCAAAAAGTCA	For qRT-PCR of UBH1
UBH1 qRT-R	CGAATCGCCGTTAGGGGTGTCTG	
FgWING27oe_GFP_F	TTTCGTAGGAACCCAATCTTCAAAAACACCGTCCTTGACTTCAACACAG	For the construction of Fgwing27oe-GFP

FgWING27oe_GFP_R	CCCGGTGAACAGCTCCTCGCCCTTGCTCACGAGTCCCTGGTTGTTCCAATTGTAG	strain
FgWING27oe_FLAG_F	TTTCGTAGGAACCCAATCTTCAAAATGGATCCACCATTTCCTGG	For the construction Fgwing27oe-FLAG strain
FgWING27oe_FLAG_R	TTTATAATCACCGTCATGGTCTTTGTAGTCGAGTCCCTGGTTGTTCCAATTGTAG	
fg01306 qRT-F	GGCTTCTTCTTCCTTCCGAGACTC	For qRT-PCR of FGSG_01306
fg01306 qRT-R	GGTGGTGGGAAGGCGAACATA	
fg10066 qRT-F	GTCGTAGGCGTTCACTACCGTGTC	For qRT-PCR of FGSG_10066
fg10066 qRT-R	GGATCTTGTATGTTCGGTACTCGTCG	
fg13697 qRT-F	AATTGCCAAAACGAGAACTCCCTA	For qRT-PCR of FGSG_13697
fg13697 qRT-R	CTGTGTAATCACTGAGCGAGACCAA	
fg10067 qRT-F	GAAATTCCTCCTTCCCGTTCTGTC	For qRT-PCR of FGSG_10067
fg10067 qRT-R	TACTCGCTCGTCCCACTCCTCAG	
fg17056 qRT-F	TATTCCTTCAATTCTTCGAGCAGCA	For qRT-PCR of FGSG_17056
fg17056 qRT-R	GGACTTTGCTAGCTGCGTCTTGAC	
fg10239 qRT-F	TGAGCGCCTCATCTACGGTGTAT	For qRT-PCR of FGSG_10239
fg10239 qRT-R	CCGTCAAAGACACCAAAGAAAGACA	
fg17056 chip-F-1	AAACTACACTGTCTCGGTCTGTGCC	For ChIP-qPCR of fg17056-1
fg17056 chip-R-1	TGAAGAGCAGAACCTTTCGTGACA	
fg17056 chip-F-2	TACAAGCCGTCATAGCACTCTCGT	For ChIP-qPCR of fg17056-2
fg17056 chip-R-2	CATTGCATACATCAGCTGAGGACAT	
fg17056 5F	ACAGGCTCTACAAGAGTGGGCTACA	For the construction of *Fg17056* deletion mutant
fg17056 5N	CCACATACTTCTGAAACAACGCTCA	
fg17056 5R	gcacaggtacacttgtttagagGAATAACAGGAAGGAAGGAAGGAGG	
fg17056 3F	ccttcaatatcatcttctgtcgTGTAGGATGGGTTTATGTGAGGCTT	
fg17056 3N	TTGGGTACAGACTTTGAGGGATTTG	
fg17056 3R	AGCCATTTGCCTCTACATTCTCCA	

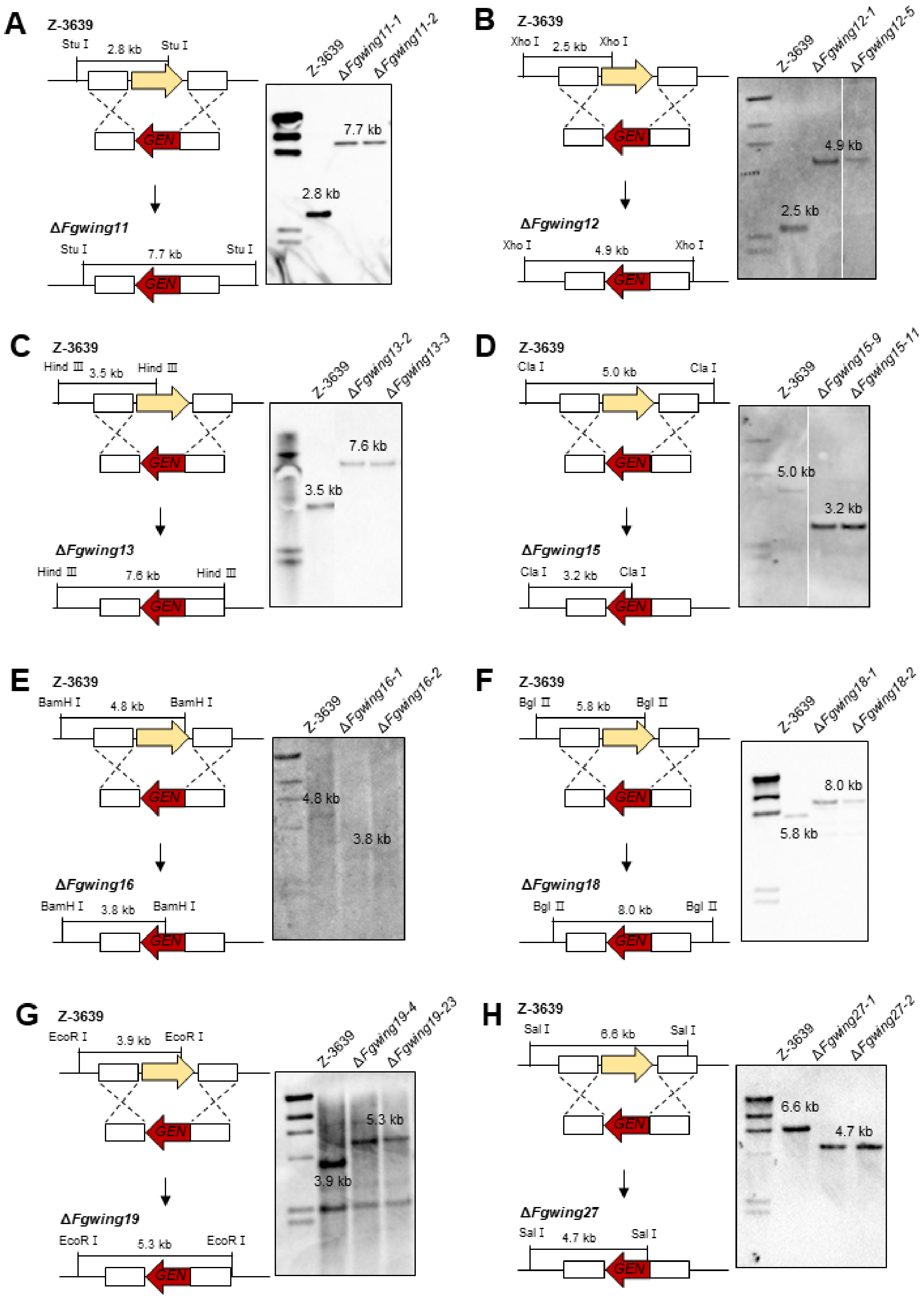

Fig. S1. Southern blot analysis to confirm the mutant construction. (A-H) The strategy for genetic manipulation is shown on the left panel, and a representative Southern blot is shown on the right panel. *GEN,* geneticin resistance gene cassette.

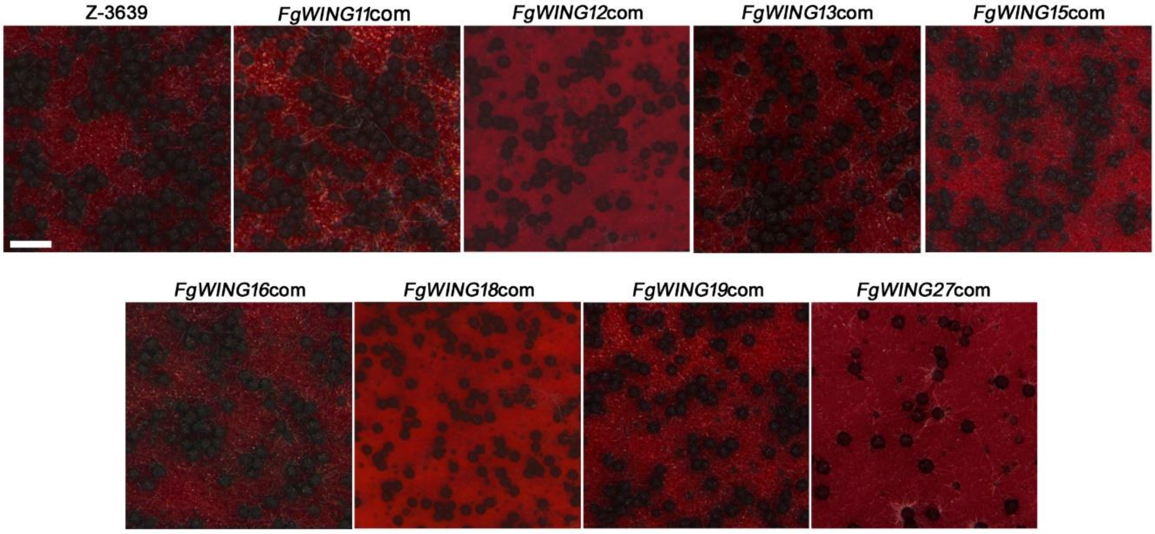

Fig. S2. Sexual development of the wild-type and *FgWING* complemented strains. Perithecium formation was observed 8 days after sexual induction. Scale bar, 500 μm.

References

1. Son H, Seo Y-S, Min K, Park AR, Lee J, Jin J-M, Lin Y, Cao P, Hong S-Y, Kim E-K, Lee S-H, Cho A, Lee S, Kim M-G, Kim Y, Kim J-E, Kim J-C, Choi GJ, Yun S-H, Lim JY, Kim M, Lee Y-H, Choi Y-D, Lee Y-W. 2011. A phenome-based functional analysis of transcription factors in the cereal head blight fungus, *Fusarium graminearum*. PLoS Pathog 7:e1002310.
2. Min K, Son H, Lim Jae Y, Choi Gyung J, Kim J-C, Harris Steven D, Lee Y-W. 2014. Transcription factor RFX1 is crucial for maintenance of genome integrity in *Fusarium graminearum*. Eukaryot Cell 13:427-436.
